# From Gut Homeostasis to Colorectal Cancer: Spatial and Temporal Reprogramming of Microbial Inosine Signaling

**DOI:** 10.3390/biomedicines14051065

**Published:** 2026-05-07

**Authors:** Lorenzo Tomassini, Teresa Pacifico, Giovanni Monteleone, Carmine Stolfi, Federica Laudisi

**Affiliations:** 1 Department of Systems Medicine, University of Rome “Tor Vergata”, 00133 Rome, Italy; lorenzo.tomassini@couldsa.uniroma2.eu (L.T.); teresa.pacifico@uniroma2.it (T.P.); gi.monteleone@med.uniroma2.it (G.M.); carmine.stolfi@uniroma2.it (C.S.); 2 Gastroenterology Unit, Policlinico Universitario Tor Vergata, 00133 Rome, Italy

**Keywords:** gut microbiota, microbial metabolites, microbiota–host interactions, intestinal homeostasis, adenosine, purine, colitis-associated cancer, tumor microenvironment, immunotherapy, T cell activation

## Abstract

Microbial metabolites are increasingly recognized as critical regulators of gut homeostasis, mediating the interaction among gut microbiota, host tissue, and immune response. Among them, inosine, which was previously considered just a byproduct of the adenosine catabolism, was recently discovered as an important bioactive purine metabolite with distinctive context-dependent signaling function. Indeed, inosine supports intestinal barrier integrity and modulates the immune response under physiological conditions. However, this scenario completely changes when chronic inflammation, dysbiosis, and colorectal cancer (CRC) develop, leading to a profound alteration of its spatial distribution and biological function. This review summarizes the biochemical properties, signaling, and sources of inosine and its role in the maintenance of gut homeostasis. We will also discuss the dynamic regulation of host–microbiota interaction, driven by inosine during CRC development and its progression following its spatial redistribution and temporal reprogramming. In particular, we will describe how inosine can shift from a tumor-supportive role to a trigger of anti-tumor immunity by promoting T cell function and macrophage polarization, becoming a critical modulator of host–microbiota crosstalk in health and disease and a promising therapeutic target for microbiome-based strategies and combined clinical approaches.

## 1. Introduction

The maintenance of gut homeostasis relies on the interplay among host tissues, the immune system, and the gut microbiota. Within this complex ecosystem, microbial metabolites play an important role as mediators of host–microbe interaction, supporting epithelial integrity, immune tolerance, and metabolic balance [1,2]. While short-chain fatty acids, bile acids, and tryptophan derivatives have been extensively studied, purine metabolites have received limited attention despite their central role in cellular metabolism and immune regulation. Among them, inosine has always been considered a byproduct of adenosine degradation. However, accumulating evidence suggests that inosine is a bioactive metabolite with context-dependent signaling functions.

Purine metabolism plays a very important role at the interface among energy homeostasis, nucleotide turnover, and immune response [3,4]. In the gut, purines are produced by both host and microbial sources, generating a dynamic extracellular purine pool. Inosine, which is mainly produced through the deamination of adenosine, represents a critical intermediate in this context. Its production may be modulated by high metabolic demand, hypoxia, inflammation, or tissue damage [5]. Unlike adenosine, which exerts important immunosuppressive function, inosine displays a broader biological profile. Thanks to its chemical stability and receptor-binding properties, it can modulate purinergic signaling over longer spatial and temporal circumstances, particularly in the gut, targeting microbial activity, epithelial barrier function, and immune response.

As stated previously, gut microbiota can be considered an important source of inosine since specific commensal bacteria are able to generate this metabolite, thereby influencing the host’s purine pool. Under gut homeostasis, microbial inosine is largely compartmentalized within the intestinal lumen. However, following barrier damage, chronic inflammation, or tumor-associated epithelia remodeling, inosine can translocate from the lumen to the tissue, allowing this metabolite to interact with immune and stromal cells in the lamina propria, with consequent alteration of the host’s immunometabolic response [6]. Indeed, the effects of inosine are highly context-dependent and mainly influenced by receptor expression, cytokine availability, and metabolic state, leading to several biological functions in health and disease.

In this review, we will summarize current knowledge about inosine biology and discuss how its biological function may be spatially and temporally reprogrammed under pathological conditions, particularly during the transition from gut homeostasis to colorectal cancer (CRC).

## 2. Methodology

A comprehensive literature search was performed using PubMed to identify relevant studies on inosine and its role in gut homeostasis and CRC, covering publications from database inception to February 2026. The search strategy combined terms related to inosine and purine metabolism (e.g., “inosine”, “purine metabolism”) with keywords associated with intestinal physiology and cancer, including “gut microbiota”, “gut dysbiosis”, “intestinal barrier”, “immune response”, “colorectal cancer”, “tumor microenvironment”, and “immunotherapy”. Studies were screened based on title and abstract for relevance, followed by full-text evaluation when appropriate. Eligible studies included peer-reviewed articles published in English that provided mechanistic or functional insights into inosine biology, including its sources, signaling pathways, and effects on epithelial and immune compartments. Both preclinical (in vitro and in vivo) and clinical studies were considered. Given the limited availability of CRC-specific clinical data, preclinical studies were prioritized for mechanistic interpretation, while clinical evidence was used to support translational relevance.

Studies were excluded if they did not directly address inosine or lacked mechanistic relevance. Review articles were considered selectively when they provided substantial conceptual insight. The reference lists of included articles were also manually screened to identify additional relevant studies. When CRC-specific data were limited, studies from related conditions (e.g., inflammatory bowel disease or experimental colitis models) were included to better contextualize the role of inosine in inflammation-associated tumorigenesis.

## 3. Role of Inosine in Gut Homeostasis

### 3.1. Chemical Structure and Signaling Pathway

Inosine is a purine nucleoside generated by the linkage of hypoxanthine to β-D-ribofuranose through an N9-glycosidic bond. It is mainly produced through the irreversible deamination of adenosine, catalyzed by adenosine deaminase (ADA). Inosine has a molecular formula of C_10_H_12_N_4_O_5_ and a molecular weight of 268.23 g/mol, making it a highly polar molecule that displays good aqueous solubility and chemical stability under physiological conditions. Inosine acts as an amphoteric compound, exhibiting weak basic properties via its nitrogen-containing heterocycle and weak acidic behavior through hydroxyl and carbonyl moieties. This dual character makes it stable at neutral pH, while it is susceptible to hydrolysis in acidic environments and to oxidative degradation or ring-opening reactions under alkaline conditions, ultimately yielding xanthine and uric acid [7].

Although inosine is not a strong nucleophile, its close structural similarity to adenosine allows it to interact with adenosine receptors, particularly the adenosine A2A subtype [8]. Specifically, it can interact with the adenosine receptor subtypes A1, A2A, A2B, and A3, a family of G protein-coupled receptors broadly expressed across immune, endothelial, epithelial, and neuronal compartments [9]. Among these, the A2A receptor exhibits the greatest functional sensitivity to inosine and mediates many of its immunomodulatory and cytoprotective effects. Engagement of both A2A and A2B receptors leads to intracellular cyclic adenosine monophosphate (cAMP) accumulation and activation of downstream protein kinase A signaling, resulting in suppression of proinflammatory cytokine production and immune activation, as well as protection from ischemic injury [10]. Inosine can also interact with the A3 receptor, a pathway that has been linked to neuroprotective effects and emerging anti-tumor activities [11].

### 3.2. Sources and Regulation of Inosine Availability

In mammalian cells, inosine is primarily generated through ADA-dependent deamination of adenosine in immune, endothelial, and epithelial cells [12,13]. This process occurs constitutively during nucleotide turnover and it is enhanced under inflammatory, ischemic, or hypoxic conditions, where increased ATP degradation leads to extracellular adenosine accumulation. In this context, inosine acts as a less reactive molecule able to limit purinergic signaling, thereby modulating the intensity and duration of adenosine receptor activation [14,15].

**Figure 1:**
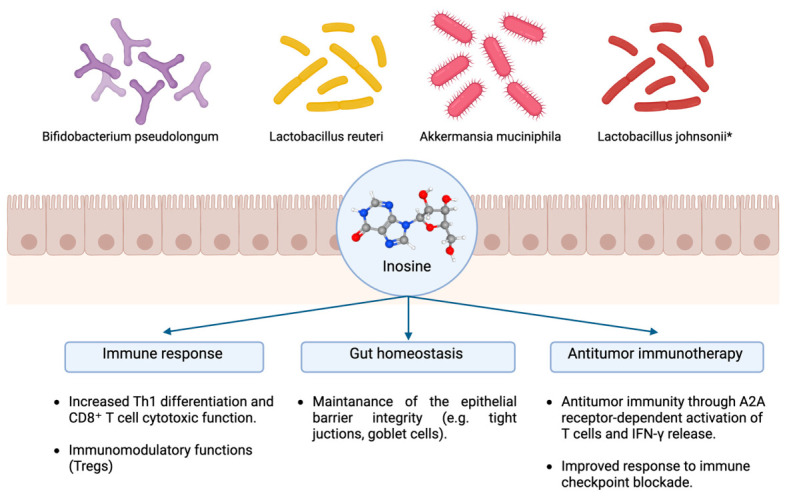
Microbial-derived inosine downstream effects. Schematic representation of the effects of microbial-derived inosine on the host’s gut homeostasis and immune response. *, indirect production of inosine resulting from adenosine degradation. Created in BioRender. https://BioRender.com/l5kq5hd (accessed on 20 April 2026). Abbreviations: Th1: T helper 1; Tregs: Regulatory T cells; IFN-γ: interferon γ.

In parallel, the intestinal microbiota represents a significant source of inosine, with specific bacterial taxa able to regulate its production (Figure 1). In particular, *Bifidobacterium pseudolongum* has been identified as a critical inosine producer, promoting anti-tumor immunity through A2A receptor-dependent activation of T cells and increased IFN-γ release, thereby improving responsiveness to immune checkpoint blockade [16]. Consistent with this, intestinal *Bifidobacterium* spp. are highlighted as relevant inosine-producing bacteria in preclinical immune checkpoint inhibitor (ICI) models. Additional studies indicate that inosine links gut microbiota composition to host immunometabolic states. In lupus models, fecal microbiota transplantation enriches *Lactobacillus* spp. (including *L. reuteri* and *L. johnsonii*), increases fecal inosine and ameliorates disease, with *L. johnsonii*-derived inosine directly suppressing B cell differentiation via ERK–HIF-1α signaling [17]. Probiotic *L. reuteri* has also been reported to restore inosine and reduce Th1/Th2 cells, supporting broader *Lactobacillus*–inosine immunomodulation [17,18]. Interestingly, *Akkermansia muciniphila*, even if it does not act as a primary inosine producer, plays an important indirect role by reinforcing epithelial barrier integrity and shaping the metabolic interactions that determine inosine availability within the intestinal niche [19]. In addition, *Lactobacillus johnsonii* has been reported to metabolize adenosine under hypoxic conditions, thereby contributing to systemic inosine pools and promoting immune-stimulatory responses in preclinical models [16]. Beyond these well-characterized taxa, several other commensal bacteria express ADA and purine nucleoside phosphorylase enzymes that contribute to intestinal inosine homeostasis [20].

Beyond single species, diverse gut commensals across *Bacillota*, *Fusobacteriota*, and *Pseudomonadota* harbor anaerobic purine-degradation gene clusters and use purines (including uric acid and adenine) as carbon and energy sources, positioning them as key regulators of luminal purine and inosine availability [20]. Additional work in hyperuricemia and gout shows that several lactic acid bacteria and *Streptococcus thermophilus* strains efficiently degrade inosine and hypoxanthine and lower serum uric acid in vivo [21].

Circulating inosine levels can be further sustained by endogenous nucleotide catabolism in metabolically active organs, such as the liver, brain, and immune system, as well as by dietary purines derived from meat, fish, and fermented foods.

Taken together, these observations indicate that systemic inosine levels reflect a composite signal integrating host metabolic status, microbial activity, and environmental inputs, making inosine a central regulator of immunometabolic balance.

### 3.3. Luminal Inosine and Epithelial Function

At physiological levels, microbiota- and host-derived inosine can support epithelial barrier integrity and mucosal balance. For instance, in vivo experimental studies show that barely leaf supplementation can promote the release of microbiota-derived inosine, as well as improve the intestinal barrier integrity [22]. Mechanistically, inosine has been reported to signal through the adenosine A2A receptor expressed on colonic epithelial cells, triggering downstream activation of peroxisome proliferator-activated receptor (PPAR) γ signaling pathways and enhancing the expression of tight-junction-associated proteins [22]. Moreover, administration of exogenous inosine displays similar effects compared to barley leaf intake [22]. In addition, in animal models of dextran sodium sulfate (DSS)- and 2,4,6-trinitrobenzene sulfonic acid (TNBS)-induced colitis, exogenous or microbiome-derived inosine can reduce weight loss, histologic injury, and mortality, as well as suppress the release of proinflammatory cytokines (e.g., TNF-α, IL-6, and IL-1β), decrease oxidative stress markers (e.g., myeloperoxidase (MPO)/malondialdehyde (MDA)) and modulate NF-κB and Nrf2 signaling pathways. Furthermore, inosine upregulates anti-oxidative enzymes, such as superoxide dismutase (SOD) and glutathione peroxidase (GSH-Px) and tight-junction proteins (e.g., zonula occludens (ZO-1), occludin, and claudin), thus improving barrier integrity and limiting bacterial translocation and consequent activation of the immune response [23,24,25]. Moreover, treatment with inosine prevents the decrease of goblet cell number and function, as well as muscular layer thickness [22]. While these observations suggest a protective role for inosine in intestinal injury, it is important to note that these studies employ animal models of chemical-induced colitis, which do not fully recapitulate the complexity and regulatory dynamics of human gut physiology.

### 3.4. Inosine-Mediated Immune Tolerance in the Gut

Beyond its effect on the epithelial compartment, inosine has emerged as a modulator of mucosal immune responses. Under pathological conditions, microbiota-derived inosine modulates T cell activity through the adenosine A2A receptor, enhancing CD8^+^ T cell and Th1 cell effector activity, thus promoting anti-tumor immune response in preclinical models of CRC [16,26]. Moreover, inosine synergizes with PD-1 and CTLA-4 immune-checkpoint inhibitors and reduces tumor growth by increasing the frequency of tumor-infiltrating leukocytes, CD4^+^ and CD8^+^ T cells in the spleen, as well as IFN-γ levels in the tumor microenvironment [16,26].

However, inosine has also been reported to promote immune tolerance by supporting regulatory T cell expansion and increasing the expression of immunoregulatory molecules, such as CD39 and CD73, in a murine model of liver injury [27]. At the metabolic level, inosine can serve as an alternative carbon source for activated T cells under conditions of glucose limitation, sustaining proliferation and effector function and thereby influencing T cell fate decisions. In particular, the ribose subunit of inosine can provide ATP and biosynthetic precursors by entering into the central metabolic pathways and can also be used by cancer cells [28,29]. However, some types of tumors are not able to metabolize inosine, while, instead, it can be processed by CD8^+^ T cells, thus improving the anti-tumor efficacy of ICIs [28]. Indeed, inosine can relieve the tumor-imposed metabolic restriction of T cells.

In conclusion, these findings suggest that inosine exerts context-dependent immunomodulatory effects, contributing to the balance between effector and regulatory immune responses within the intestinal mucosa.

### 3.5. Loss of Balance

Inosine signaling is highly sensitive to perturbations in microbial composition, and dysbiosis or antibiotic exposure can indeed reduce microbial inosine levels and weaken epithelial integrity and immune homeostasis [22,23]. Experimental animal models indicate that decreased inosine availability due to a reduced frequency of inosine-producing bacteria taxa, such as *Lactobacillus reuteri*, fails to prolong the survival of “Scurfy” mice, which lack regulatory T cells, and to limit intestinal inflammation [18].

Although direct experimental evidence connecting inosine dysregulation to the initiation of CRC remains limited, accumulating data suggest that inosine-mediated pathways intersect with inflammation-driven processes that can influence the tumor microenvironment [16]. Thus, precise spatial and temporal regulation of inosine production and signaling appears to be important to modulate gut homeostasis and prevent pathological conditions.

## 4. Role of Inosine in CRC Development

The development of CRC is mainly driven by chronic inflammation, as well as alterations of the intestinal barrier and metabolic reprogramming of epithelial and stromal cells [30,31]. These conditions support continuous epithelial turnover and consequent tumor growth, together with new availability, localization, and biological effects of microbial metabolites [31,32]. In this context, metabolic crosstalk between cancer cells, immune cells, stromal components, and the host’s microbiota leads to the reshaping of purine metabolic pathways. For example, inosine may also acquire new functions compared to those observed under physiological conditions, depending on new spatial reorganization and disease stage [33,34].

### 4.1. Spatial Redistribution of Inosine During Colorectal Tumorigenesis

Unfortunately, studies reporting the effects of the spatial redistribution of inosine across different intestinal compartments along CRC onset are currently missing since most of the clinical and preclinical data were generated from a bulk metabolomic analysis [33,35]. Under physiological conditions, microbial inosine is confined to the intestinal lumen and its access to the underlying tissue and immune cells is tightly regulated by the intestinal epithelial barrier. In this setting, inosine-driven effects are confined to the modulation of epithelial stress and mucosal immune response, contributing to tissue protection and immune homeostasis [24,25]. During CRC progression, however, tumor tissues exhibit dysregulated expression of key enzymes involved in purine metabolism, such as ADA and ectonucleotidases, suggesting altered local production, degradation, and turnover of inosine within the tumor microenvironment [36,37]. These metabolic changes are further exacerbated by chronic inflammation, hypoxia, and disruption of epithelial barrier integrity. Loss of normal crypt–villus compartmentalization and increased permeability facilitate the translocation of luminal metabolites, including inosine, into mucosal and stromal compartments. Recent integrated kinetic and single-cell analyses indicate that purine metabolism is heterogeneously distributed among malignant, stromal, and immune cell populations, creating spatial gradients of bioactive purines [38,39]. This altered metabolic landscape may influence tumor cell behavior, immune cell activation, and stromal remodeling, with potential consequences for tumor progression and immune regulation [34]. These findings strongly suggest that CRC development is associated with a profound spatial reorganization of the purinergic signaling network.

### 4.2. Temporal Reprogramming of Inosine Signaling in CRC

Currently, no human studies have longitudinally tracked inosine levels or purine metabolic flux from colitis through dysplasia to established CRC. Consequently, any temporal model must integrate heterogeneous datasets from experimental colitis models, inflammatory bowel diseases (IBD) and colitis-associated CRC (CAC) patients, as well as tumor-stage studies. As previously mentioned, in acute and chronic experimental colitis, inosine mainly supports anti-inflammatory response, limiting immune activation and epithelial damage, thus contributing to the maintenance or trying to re-establish gut homeostasis [22,23,25]. Large-scale metabolomic studies in IBD patients reveal widespread perturbations in purine and pyrimidine metabolism and link baseline serum purine metabolites to disease severity and progression [40,41,42]. However, these studies do not resolve compartment-specific inosine dynamics nor follow patients into overt CRC development.

Over time, chronic inflammation and dysbiosis promote epithelial damage and cell death, driving persistent release of nucleotides and other metabolites that reshape immune composition, redox balance, and tumor metabolism along the inflammation–dysplasia–carcinoma axis [43,44]. This evolving microenvironment provides both metabolic substrates and signaling cues that facilitate malignant transformation and tumor progression. Recent evidence indicates that oncogenic signaling pathways can directly reprogram purine metabolism in CRC cells. A recent multi-omics study shows that TGF-β1 markedly increases the intracellular inosine levels by inducing laccase domain-containing 1 (LACC1) enzyme in SW480 cells, a multifunctional purine enzyme that converts adenosine to inosine [45]. TGF-β1-driven upregulation of LACC1 is essential for inosine accumulation. Moreover, genetic knockdown of LACC1 reduces inosine levels and attenuates TGF-β1-mediated epithelial to mesenchymal transition (EMT) markers and motility (e.g., E-cadherin, N-cadherin, vimentin, and tight-junction components) [45]. In parallel, independent work demonstrates that multiple cancer cell types, including SW480 and HT29 cells, can use inosine as an alternative carbon source under glucose-restricted conditions [28]. This metabolic flexibility enhances mitochondrial respiration and survival of tumor cells in nutrient-poor tumor regions [28].

Thus, these findings suggest a temporal shift in inosine function during CRC development, from a homeostatic anti-inflammatory mediator in early disease, to a tumor-supportive metabolite in advanced stages, by promoting the EMT-driven invasion and serving as a metabolic substrate for cancer cell adaptation to nutrient deprivation.

### 4.3. Inosine-Mediated Modulation of Anti-Tumor Immunity in CRC

Despite evidence supporting a pro-tumorigenic role of inosine in CRC cell metabolism and invasion, accumulating data suggest that inosine can also promote anti-tumor immune response, highlighting its dual and context-dependent nature.

As described previously, inosine released by the gut commensal *Bifidobacterium pseudolongum* significantly enhances the efficacy of immune checkpoint blockade therapy. In germ-free mice bearing transplantable MC38 colon cancers, as well as in models of mismatch repair-deficient (MMRD) small intestinal cancer or mismatch repair-proficient (MMRP) colon cancer, oral inosine supplementation improved responses to CTLA-4 and, to a lesser extent, PD-1 blockade [16]. In particular, it activates T cells through the engagement of the adenosine A2A receptors, promoting the Th1 anti-tumor response, characterized by increased IFN-γ production [16]. Beyond adaptive immunity, inosine may also influence myeloid cell behavior within the CRC microenvironment. In mice bearing CT26 cells, inosine can also promote the polarization of macrophages toward an M1-like proinflammatory phenotype by increasing CD86 and inducible nitric oxide synthase (iNOS), thus restricting tumor growth and preventing the spread of metastasis [46].

**Figure 2:**
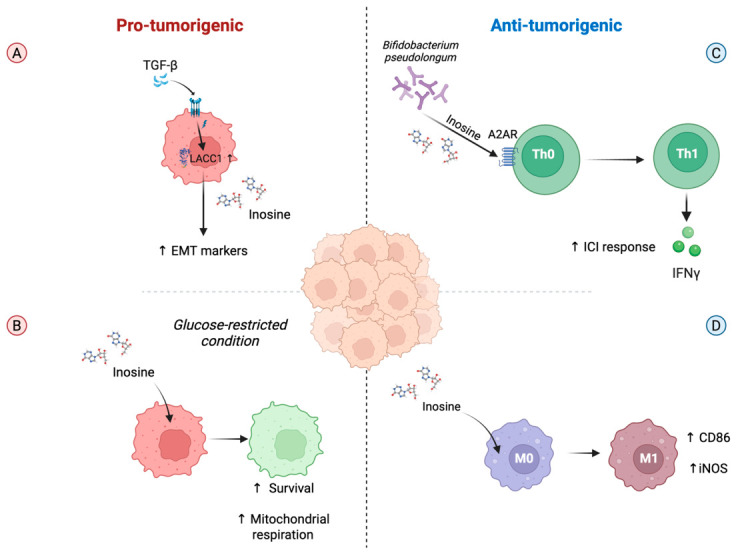
Pro- and anti-tumorigenic effects of inosine in colorectal cancer (CRC). In CRC cells, TGF-β1 increases inosine levels by inducing LACC1 enzyme that converts adenosine to inosine, which in turn promotes mesenchymal transition markers and motility (**A**). Moreover, inosine can be used as an alternative carbon source by CRC cells under glucose-restricted conditions (**B**). However, inosine can also exert anti-tumorigenic effects by promoting the differentiation of Th1 cells, thus increasing the response to immune checkpoint inhibitors (**C**). In addition, inosine can also promote the polarization of macrophages toward an M1-like proinflammatory phenotype (**D**). Created in BioRender. https://BioRender.com/m6rtv2r (accessed on 20 April 2026). Abbreviations: TGF-β: Transforming growth factor-β; LACC1: laccase domain-containing 1; EMT: epithelial–mesenchymal transition; A2AR: A2A receptor; Th0: T helper 0; Th1: T helper 1; ICI: Immune checkpoint inhibitors; IFN-γ: Interferon-γ; M0: Macrophage M0; M1: Macrophage M1; iNOS: inducible nitric oxide synthase.

Collectively, these findings indicate that inosine exerts complex and opposing effects in CRC since it supports tumor cell metabolism and migration (i.e., by promoting mesenchymal transition markers and motility or representing an alternative carbon source by CRC cells under glucose-restricted conditions), while it enhances the anti-tumor immune response (i.e., by supporting Th1 cell differentiation or macrophage M1 polarization) (Figure 2). The balance between these two functions may depend on disease stage, spatial organization, or composition of the tumor microenvironment.

### 4.4. Targeting the Inosine Axis to Rebalance Intestinal Immunity and Improve Therapeutic Outcomes in CRC

Despite important experimental and preclinical evidence for microbiota-metabolite control of CRC, inosine remains under-characterized in CRC patients compared to other microbial metabolites (e.g., bile acids, short-chain fatty acids). Indeed, only one phase-2 clinical trial has evaluated inosine as an adjunct to immunotherapy in a mixed advanced solid-tumor population, including CRC [47]. In particular, a total of 172 patients were randomly assigned to two groups, one receiving the PD-1/PD-L1 inhibitor and chemotherapy (*n* = 86) and one treated with the PD-1/PD-L1 inhibitor, chemotherapy, plus inosine (0.2 g orally three times/day, *n* = 86) [47]. The results reported that targeted combination therapy with inosine is able to mildly enhance the efficacy of the ICI and reduce severe toxicity [47]. However, CRC-specific results were not reported separately (*n* = 10) but were included in the group of malignant tumors of the digestive system (*n* = 55).

Recent experimental evidence highlights the possibility to modulate inosine production by employing engineered microbes. For example, engineered probiotics can degrade adenosine into inosine, thus limiting immunosuppression and increasing T cell infiltration, which contributes to reducing CRC growth in an orthotopic CRC mouse model [48]. This approach successfully increases local inosine availability, as well as impairs immune suppression driven by adenosine in the tumor microenvironment.

All this evidence suggests that inosine may be considered an example of a “microbial medicine”, illustrating how microbiota-derived metabolites can contribute to the modulation of anti-tumor immunity and potentially influence the efficacy of cancer therapies. Beyond its role as a metabolic intermediate, inosine has been shown in preclinical models to affect T cell function, macrophage polarization, and the broader tumor microenvironment. These observations place inosine within a broader and emerging class of microbiome-derived therapeutic strategies [49,50]. However, the use of inosine-producing probiotics is limited by challenges in selecting strains, maintaining stability, and achieving effective colonization within the host microbiota [51]. Moreover, the efficiency of strains to produce inosine in vivo can vary greatly based on the patient’s baseline microbiota, diet, and environmental influences. Additionally, safety issues, especially for immunocompromised individuals, along with regulatory obstacles for engineered microbial therapies, are important concerns. In addition, systemic inosine supplementation presents challenges in terms of pharmacokinetics, optimal dosing, and potential off-target effects due to limited clinical data. Indeed, further studies in clinical settings are required to better define the therapeutic relevance of inosine and to determine how microbiota-derived metabolites can be effectively and safely integrated into cancer treatment strategies.

## 5. Conclusions

**Figure 3:**
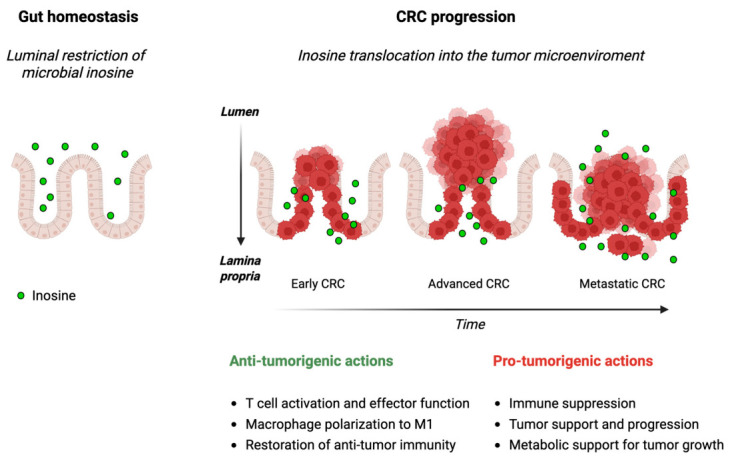
Spatial and temporal reprogramming of microbial inosine. Spatial redistribution and temporal reprogramming of microbial inosine during gut homeostasis and CRC progression, with related anti-tumorigenic and pro-tumorigenic driven effects. Created in BioRender. https://BioRender.com/2rjs9hh (accessed on 26 April 2026). Abbreviations: CRC, colorectal cancer.

Microbial inosine is now increasingly recognized as a crucial metabolite that links gut microbial activity to purine metabolism, epithelial integrity, immune response, and CRC progression. Luminal inosine supports gut homeostasis, but dysbiosis, chronic inflammation and tumorigenesis can deeply alter its spatial distribution and signaling (Figure 3). These changes confer on inosine both pro-and anti-tumorigenic effects, highlighting its context-dependent nature. This spatial and temporal plasticity makes this metabolite a critical modulator of host–microbiota crosstalk in health and disease and a promising therapeutic target for microbiome-based strategies and combined clinical approaches.

## Data Availability

No new data were created or analyzed in this study. Data sharing is not applicable to this article.
